# The metabolic and proteomic repertoires of periderm tissue in skin of the reticulated Sikkim cucumber fruit

**DOI:** 10.1093/hr/uhac092

**Published:** 2022-04-22

**Authors:** Gulab Chand Arya, Yonghui Dong, Uwe Heinig, Nir Shahaf, Yana Kazachkova, Elinor Aviv-Sharon, Gal Nomberg, Ofir Marinov, Ekaterina Manasherova, Asaph Aharoni, Hagai Cohen

**Affiliations:** 1 Department of Vegetable and Field Crops, Institute of Plant Sciences, Agricultural Research Organization (ARO), Volcani Center, Rishon Lezion 7505101, Israel; 2 Department of Plant and Environmental Sciences, Weizmann Institute of Science, Rehovot 7610001, Israel; 3 Department of Life Sciences Core Facilities, Weizmann Institute of Science, Rehovot 7610001, Israel; 4 Department of Plant Pathology and Microbiology, Robert H. Smith Faculty of Agriculture, Food and Environment, The Hebrew University of Jerusalem, Rehovot 7610001, Israel

## Abstract

Suberized and/or lignified (i.e. lignosuberized) periderm tissue appears often on surface of fleshy fruit skin by mechanical damage caused following environmental cues or developmental programs. The mechanisms underlying lignosuberization remain largely unknown to date. Here, we combined an assortment of microscopical techniques with an integrative multi-omics approach comprising proteomics, metabolomics and lipidomics to identify novel molecular components involved in fruit skin lignosuberization. We chose to investigate the corky Sikkim cucumber (*Cucumis sativus* var. *sikkimensis*) fruit. During development, the skin of this unique species undergoes massive cracking and is coated with a thick corky layer, making it an excellent model system for revealing fundamental cellular machineries involved in fruit skin lignosuberization. The large-scale data generated provides a significant source for the field of skin periderm tissue formation in fleshy fruit and suberin metabolism.

## Introduction

In fleshy fruit, periderm tissue often forms above the outer skin epidermal layer upon mechanical damage to the skin caused by abiotic (*e.g.* rain, wind) or biotic (*e.g.* herbivory, pathogen attack) insults [1]. It can also arise as part of a coordinated wound-induced process when fruit skin development is hampered. This specialized coating typically appears as a brown, rough corky matrix above the skin. Often referred to as “russeting” [2] or “reticulation” [3], this phenomenon occurs on a range of fruit, such as apple (*Malus x domestica*), pear (*Pyrus communis*) and melon (*Cucumis melo*) varieties. The formation of lignosuberized periderm tissue holds agronomical and physiological significance as it maintains the integrity of the skin surface, and protects the growing and expanding fruit under skin-failure circumstances [4]. Previous studies established that reticulation impacts melon fruit skin firmness and elasticity [5, 6, 7], maintains the turgor pressure and waterproofing volume of hypodermal cells lying beneath the skin layer [8], and delivers enhanced tolerance against mechanical injury [9]. Similarly, newly-formed phellem cells that build the russeted coating in apple fruit were shown to replace damaged epidermis cells, thereby preserving normal water loss during fruit development and post-harvest [2, 10].

The fruit skin is typically made of epidermal cell layer covered by cuticle that is largely composed of the cutin polymer made of C_16_ and C_18_ ω-hydroxylated fatty acids, and very long chain fatty acid (VLCFA)-derived epicuticular waxes [11, 12]. As noted above, once a periderm tissue is formed below the surface of a fleshy fruit it changes vividly the morpho-physiological properties of the skin. Nevertheless, the chemistry of the fruit skin is another aspect that is significantly altered upon the formation of periderm that unlike the cuticle, is built from the suberin polymer consisting of C_20_ to C_26_ VLCFA derivatives, phenolics and glycerol-based alkyl ferulates [13]; in addition to lignin, a complex aromatic polymer derived mainly from the three hydroxycinnamyl monomers *p*-coumaryl, coniferyl and sinapyl alcohols [14]. Both the suberin and lignin pathways tightly depend on the flow of aromatic components, supplied by the core phenylpropanoid pathway [15].

In the current study, we investigated fruit of the Sikkim cucumber (*Cucumis sativus* var. *sikkimensis*), a native cultivar of the Himalayas, whose skin undergoes massive cracking during development that resembles skin damage phenotypes in other reticulated fruit species. We employed advanced microscopy, metabolomics and proteomics to characterize the structural, metabolic and proteomic changes that occur in the fruit skin at early and late developmental stages. Our findings highlight morphological and structural attributes, prevalent metabolic repertoires, and key proteins concomitant with skin lignosuberization processes in fleshy fruit.

## Results

### The Sikkim cucumber fruit undergoes massive skin cracking during development

**Figure 1:**
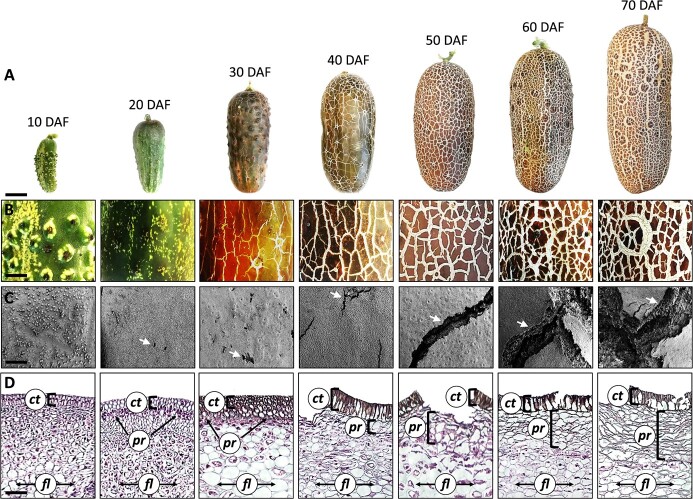
The Sikkim cucumber fruit undergoes massive skin cracking during development. (A) Phenotypes of the Sikkim cucumber fruit along development. DAF, days after fertilization. Scale bar = 2 cm. (B) Light microscopy images of fruit surfaces showing skin cracking patterning along fruit development. Scale bar = 5 mm. (C) Scanning electron microscopy (SEM) images displaying the fine structure of fruit skin surfaces. White arrowheads indicate cracked regions. Scale bar = 300 μm. (D) Light microscopy images of skin cross-sections along fruit development. Sections were counterstained with Hematoxylin and Eosin. Abbreviations: *ct*, cuticle; *pr*, peridermal layer; *fl*, flesh cells. Scale bar = 400 μm.

We performed microscopic investigation of seven stages along the Sikkim cucumber’s fruit development at ten-day intervals; from 10 days after fertilization (DAF), when the immature fruit is green with no visible skin cracking, to 70 DAF, when the fully-expanded mature fruit is covered with a thick corky brownish skin decorated by large cracks (Fig. 1A-B). At 20 DAF, we detected the first signs of brown patches on the skin surrounding the pedicel, but no cracks were observed. Yet, by 30 DAF, cracks formed in these regions and the brown coating covered the entire fruit skin. From this stage until full maturity, the extent of cracking increased and the fractures widened and deepened (Fig. 1A-B).

Scanning electron microcopy (SEM) observations revealed more details with respect to fruit skin cracking along development. At 10 DAF, the skin was densely covered with trichomes, which disappeared by 20 DAF, at which point the skin displayed some micro-cracks, though they were not visible under light microscopy (Fig. 1C). As the fruit further matured, these cracks gradually widened, with a large mass of cells bursting from the inner part of the fruit observed at 60 and 70 DAF, resulting in the elevation of typical cuticle-coated epidermal cells (Fig. 1C). Cross-section SEM micrographs revealed typical organized phellem cell layers (Supplementary Fig. S1), as previously reported for wound periderm tissues of potato tubers [16, 17].

Histological cross-sections of skin tissues counterstained with Hematoxylin and Eosin confirmed the formation of a typical wound periderm beneath the outermost epidermal cell layer. Periderm tissue consisting of 1–2 cell layers was detected at 20 DAF, coinciding with the first signs of a brownish corky matrix above the skin (Fig. 1D). The cuticle-coated epidermal cell layer at 10, 20 and 30 DAF was still intact, even though some small micro-cracks appeared on the skin of 30 DAF fruit (Fig. 1C-D). From 40 DAF until full maturity, the periderm layer expanded below the cuticle towards the flesh cells, gradually forming more and more cell layers, while major cracks that disrupted the cuticle’s discontinuity could be detected (Fig. 1B-D). Taken together, these observations suggest a link between skin cracking in the Sikkim cucumber fruit and the formation of a typical wound periderm tissue.

### Skin cracking in the Sikkim cucumber fruit is made of a lignosuberized wound periderm

**Figure 2:**
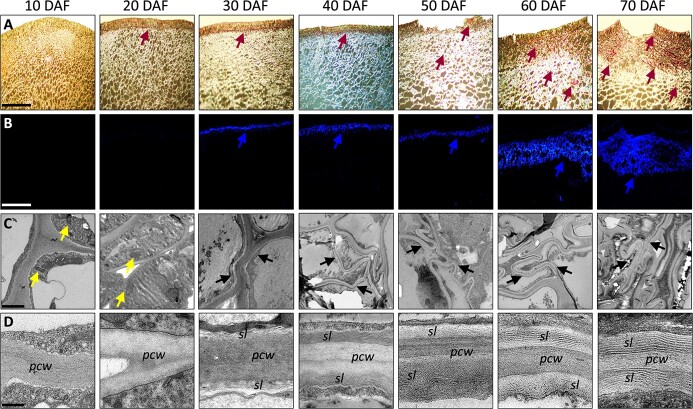
Skin cracking in the Sikkim cucumber fruit is made of a lignosuberized wound periderm. (A) Light microscopy images of histological skin sections stained for lignin by phloroglucinol-HCL. Purple arrow heads point at lignified cells that are stained by purple coloration. Scale bar = 100 μm. (B) Fluorescent microscopy images of histological skin sections stained for suberin by fluorol yellow and observed under 488 nm filter. Blue arrowheads point at suberized cells that are stained by blue coloration. Scale bar = 100 μm. (C) Transmission electron microscopy (TEM) images displaying the formation of suberin lamellae. Yellow and black arrowheads point at chloroplasts and suberin lamellae, respectively. Scale bar = 2 μm. (D) TEM images displaying primary cell walls (*pcw*) and suberin lamellae (*sl*). Scale bar = 30 nm.

The formation of a wound periderm in fleshy fruit has been associated with the metabolism of lignin and suberin. To further assess whether the periderm beneath Sikkim cucumber fruit regions with cracked skin is lignified and/or suberized, we exploited several microscopical approaches. First, we stained skin sections with phloroglucinol-hydrochloric acid (PG-HCL), whose interaction with cinnamaldehyde end-groups of lignin generates a red-violet color. The first signs of lignin were detected already at 20 DAF, in the cell layer beneath the fruit cuticle (Fig. 2A). The lignification seems to gradually expand toward the inner skin cells between 20 and 50 DAF, a period also marked by the formation of cracks and suberin lamellae. However, at the mature developmental stages, i.e. 60 and 70 DAF, the lignified layer beneath the cracked regions is comprised of many cells (Fig. 2A). Remarkably, we detected lignin also in conical cells building the cuticle of 50, 60 and 70 DAF fruit, suggesting that lignin deposition might also occur in skin cuticle specifically in cracked regions.

Next, sections were stained with Fluorol Yellow (FY), a specific suberin fluorescent dye. In accord with the periderm tissue patterning observed by histology, suberin was confined to cells building the wound periderm underneath the injured cuticle-coated epidermal cell layer. No FY fluorescent signals could be detected in 10 and 20 DAF skin samples, but they were observed in 30 DAF skin, which subsequently spread toward the lower cell layers, particularly at 60 and 70 DAF (Fig. 2B). These results further indicate that when skin damage and cuticle fractures become more severe, the wounded periderm tissue rapidly expands inwardly into the flesh, where it deposits additional suberized cell layers (Fig. 2B).

Next, we studied the deposition pattern of suberin lamellae in cells building the periderm tissue by examining ultrathin cross-sections of skin under transmission electron microscopy (TEM). A typical thick primary cell-wall structure was detected in the layer of cells beneath the cuticle at 10 and 20 DAF, where we also observed chloroplasts, as expected for a green fruit skin at early developmental stages with the capacity to perform photosynthesis (Fig. 2C). At 30 DAF, however, thin, unorganized lamellar structures appeared adjacent to the primary wall of these cells (Fig. 2C-D). From 40 DAF until 70 DAF, we detected multiple cell layers featuring distinct organized suberin lamellae deposits between their plasma membrane and primary cell wall; these deposits tended to expand and widen with fruit maturation (Fig. 2C-D). Collectively, our findings indicate that a large portion of cells that form the wound periderm tissue in the Sikkim cucumber fruit is both lignified and suberized.

### Suberin is confined to cracked regions of periderm tissues

**Figure 3:**
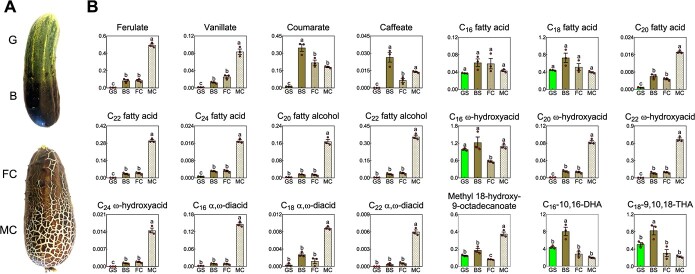
Suberin is confined to cracked regions of periderm tissue. (A) Sikkim cucumber fruit exhibiting intermediate appearances of skin spanning from green (G) skin, through skin with brownish coating (B), to skin with few cracks (FC) or many cracks (MC). (B) Levels of individual cutin and suberin monomers in de-lipidated and transesterified skin tissues dissected as in (A). Cutin and suberin profiles were analyzed by gas chromatography–mass spectrometry (GC–MS); y-axes represent relative peak areas following normalization to a C_32_ alkane internal standard. Data represent means ± SE of three biological replicates each per sample (generated from a pool of skin tissue from four different fruit). Significance was calculated according to two-way ANOVA test of *p* value <0.05.

To further understand the spatial distribution of suberin in periderm tissue, we profiled the cutin and suberin monomer composition of a range of fruit skin samples with distinct appearances: green skin (G), skin with a brownish coating (B), skin with few cracks (FC), skin with many cracks (MC), via gas chromatography–mass spectrometry (GC–MS) [3] (Fig. 3A). Using this approach, we positivity annotated 21 metabolites typically associated with either cutin or suberin polyesters belonging to the biochemical groups of hydroxycinamic and hydroxybenzoic acids, fatty acids, fatty alcohols, ω-hydroxyacids and α,ω-diacids. Among the skin samples, MC accumulated the highest amounts of ferulic and vanillic acids, which are essential for the formation of the suberin polyphenolic domain (SPPD). These two metabolites were absent from G samples and were present at relatively low levels in B and FC samples (Fig. 3B). Other detected metabolites were building blocks essential for the suberin polyaliphatic domain (SPAD); these, too, amassed in great number in MC skin, more moderately in FC and B skin, and constituted minute quantities in G skin. These metabolites include C_20_, C_22_ and C_24_ fatty acids, C_20_ and C_22_ fatty alcohols, C_20_, C_22_ and C_24_ ω-hydroxyacids, as well as C_16_, C_18_ and C_22_ α,ω-diacids. Likewise, 18-hydroxyoctadec-9-enoic acid highly accumulated in MC skin compared to all other skin types (Fig. 3B). Taken together, the appearance of many cracks on top of the fruit skin was mostly linked to the production and accumulation of typical SPPD and SPAD building blocks. In addition, the formation of either few or many cracks on top of the reticulated skin seem to cause significant reductions in the levels of cutin monomers.

An additional significant propensity we observed was the apparent overtone between the induction of suberin in the periderm and a decrease in the levels of typical cutin monomers. While C_16_ and C_18_ fatty acids displayed similar levels in all skin types, the most predominant cutin monomers, C16 ω-hydroxyacid, C_16_–10,16-dihydroxyacid and C_18_–9,10,18-trihydroxyacid, decreased significantly in the crack-containing FC and MC skins (Fig. 3B). Interestingly, all three monomers accumulated in B and G skin at relatively similar levels, suggesting that the brownish coating at the early stages of suberization does not affect the levels of cutin. Likewise, coumaric acid and caffeic acid, which are hydroxycinamic acids associated with the cutin polyester, highly accumulated in G and B skin, but then decreased upon the appearance of cracks, further strengthening the concept that the induction of suberin is at the expense of cutin monomer biosynthesis, seemingly because both polyesters share some of their precursors (Fig. 3B).

### Deciphering the metabolic repertoire of periderm tissue

**Figure 4:**
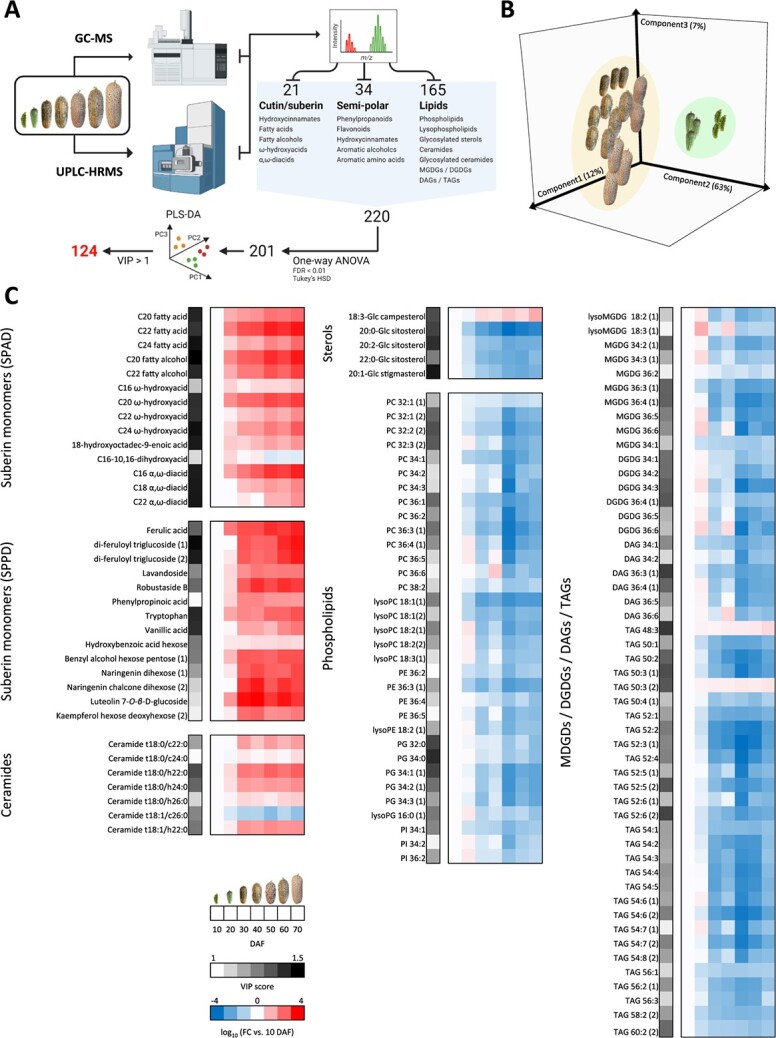
A multi-omics metabolite profiling approach to decipher the metabolic repertoire of periderm tissue. (A) Schematic of the multi-omics profiling approach: GC–MS for cutin and suberin monomers of various biochemical classes; and ultra performance liquid chromatography coupled with a high resolution mass spectrometer (UPLC-HRMS) for the identification of semi-polar metabolites (the polar phase, metabolomics) and lipids (the non-polar phase, lipidomics) from different biochemical classes. One-way ANOVA assay [based on an adjusted *p*-value (FDR) cutoff of <0.01 and post-hoc Tukey’s HSD] followed by a Partial Least Squares-Discriminant Analysis (PLS-DA) narrowed the metabolite subset to 124 metabolites. (B) PLS-DA of 201 metabolites isolated by a one-way ANOVA test as described in (A); the variances explained by each of the three components (PC1, PC2 and PC3) appear in parentheses. (C) Heatmaps representing the log_10_ fold chance (FC) in each metabolite’s level vs. its level in 10 DAF skin of the 124 metabolites isolated following one-way ANOVA and PLS-DA as described in (A). Blue colors indicate reduced levels, and red colors increased levels. Gray legend represents the variable’s importance score (VIP) according to the PLS-DA described in (A).

To examine the metabolic repertoire of skin periderm tissue formation at the temporal level, we subjected skin tissue from the seven investigated developmental stages to comparative profiling via: (i) GC–MS for cutin and suberin monomers (21 metabolites noted above); and (ii) Ultra performance liquid chromatography (UPLC) coupled to a high-resolution mass spectrometer (UPLC-HRMS) (Fig. 4A). To this end we used a protocol that allows the simultaneous isolation of semi-polar metabolites (the polar phase, metabolomics) and lipids (the non-polar phase, lipidomics). Based on in-house mass spectra and public libraries, we were able to positively annotate 34 semi-polar metabolites belonging to phenylpropanoids, flavonoids, hydroxycinnamates, aromatic alcohols and aromatic amino acids, in addition to 165 lipids belonging to phospholipids and lysophospholipids, glycosylated sterols, ceramides and their glycosylated derivatives, as well as mono- and di-galactosyldiacylglycerols (MGDGs and DGDGs, respectively) and di- and tri-acylglycerides (DAGs and TAGs, respectively) (Fig. 4A).

A one-way ANOVA assay [based on adjusted *p*-value (FDR) cutoff of <0.01 and post-hoc Tukey’s HSD] revealed that 201 of the total 220 annotated metabolites were significantly altered in abundance along fruit development (Fig. 4A). Analysis of skin samples according to this metabolic subset under a partial least squares-discriminant analysis (PLS-DA) delineated a clear bi-separation pattern, with 10 and 20 DAF skin samples grouped together, and the 30, 40, 50, 60 and 70 DAF skin samples together forming a second cluster (Fig. 4B). The PLS-DA model allows the identification of variable’s importance score (VIP). Herein, we isolated the 124 metabolites with the highest VIP scores (VIP >1), representing the most contributory factors in the PLS-DA discrimination model, and, therefore, the metabolites that mostly contributed to the separation patterns between skin samples.

Of these 124 metabolites, 36 exhibited non-detectable or very low levels in 10 and 20 DAF skin tissues, which greatly increased from 30 DAF onward, when the suberized tissue starts to form. The levels of the vast majority of these metabolites peaked at more advanced developmental stages, particularly between 50 and 70 DAF. Logically, this subset of metabolites comprised most of the typical building blocks of the SPAD, including fatty acids C_20_, C_22_ and C_24_, fatty alcohols C_20_ and C_22_, $\omega $-hydroxyacids C_16_, C_20_, C_22_ and C_24_, 18-hydroxyoctadec-9-enoic acid, as well as α,$\omega $-diacids C_16_, C_18_ and C_22_ (Fig. 4C). An additional subset of metabolites, those belonging to the SPPD class, increased in abundance upon the appearance of periderm tissue. These included the phenylpropanoid derivatives of ferulic acid, di-ferulyol triglucoside (two isomers), ferulic acid 4-*O*-*β*-D-glucopynoside (lavandoside), robustaside B (6′-3″, 4″-dihydroxycinnamoyl arbutin derivative) and phenylpropionic acid (hydrocinnamic acid); the aromatic amino acid tryptophan; hydroxybenzoic acid hexose and vanillic acid (4-hydroxy-3-methoxybenzoic acid); the aromatic benzyl alcohol hexose pentose; and several flavonoids such as naringenin dihexose, naringenin chalcone dihexose, luteolin 7-*O*-*β*-D-glucoside and kaempferol hexose deoxyhexose (Fig. 4C). The sterol glycoside 18:3-Glc-campesterol and TAGs 48:3 and TAG 50:3 showed similar trends of accumulation. A remarkable increase in abundance was detected in all identified ceramides apart from ceramide t18:1/c26:0 that exhibited decreased abundance with the formation of periderm tissue (Fig. 4C), suggesting a possible role for this class of lipids in lignosuberized periderm tissue establishment. The other 88 metabolites displayed an opposite trend, i.e. a gradual decrease in abundance with fruit development. These included various types of glycosylated sterols, phospholipids, MDGDs, DGDGs, DAGs and TAGs. In line with our earlier observation, the most abundant cutin monomer, C_16_–10,16-dihydroxyacid, gradually decreased towards fruit maturity, particularly in the 50, 60 and 70 DAF samples (Fig. 4C). Overall, these findings suggest that periderm tissue formation is principally associated with over-accumulation of suberin components, phenylpropanoids, flavonoids and ceramides, along with reduced amounts of membrane components and storage lipids.

### Proteomics of skin tissues during Sikkim cucumber fruit development

**Figure 5:**
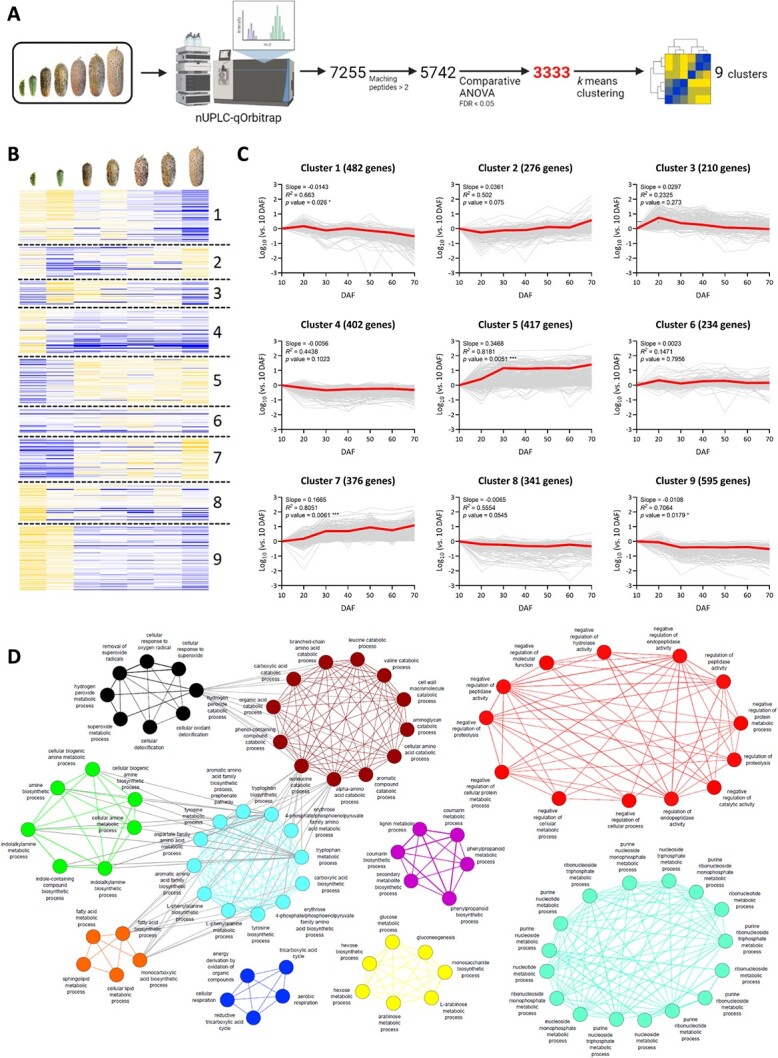
Proteomics of skin tissue during Sikkim cucumber fruit development. (A) Schematic of the proteomics profiling steps of skin tissues along fruit development. The analysis was performed using a nano-UPLC coupled with a quadruple Orbitrap mass spectrometer. 7255 putative proteins were detected based on their mass fragments, of which 5742 were identified by at least two uniquely matching peptides. Following a comparative ANOVA (FDR <0.05), the subset was narrowed down to 3333 proteins exhibiting significant changes in their levels along fruit development. Finally, a *k*-means clustering assay divided these proteins into 9 exclusive clusters based on their abundance outlines. (B) Heatmap of the relative abundances of proteins in each developmental stage (blue colors represent low abundances, and yellow colors high abundances), divided into 9 exclusive clusters following the *k*-means clustering assay described in (A). (C) Curve graphs representing the accumulation trend of proteins included in each of the 9 exclusive clusters following the *k*-means clustering assay described in (A) and (B), as the log_10_ fold change vs. their abundances in 10 DAF skin. The number of proteins, calculated slopes, *R^2^* and *p* values are presented for each cluster. Grey curves represent the accumulation trend of each individual protein included in the cluster, while bold red curves represent the averaged patterns of proteins. (D) A network-based representation of gene ontology (GO) functional terms of 793 proteins belonging to clusters 5 and 7. The GO analysis was performed using the tools embedded in the Cucurbit Genomics Database (CuGenDB; http://cucurbitgenomics.org/), and the network was constructed using Cytoscape3.9.0, an open-source platform for visualizing complex networks (https://cytoscape.org/). The different colors represent separate networks with distinct biological processes.

To obtain insight into mechanisms involved in the initiation and regulation of lignosuberized periderms, we carried out comparative proteomics profiling of skin tissues along fruit development. Overall, 7255 putative proteins were detected based on their mass fragments, of which 5742 were identified by at least two uniquely matching peptides (Fig. 5A). In order to highlight skin proteins exhibiting a significant change in abundance along fruit development, we narrowed down the proteomics subset by employing a comparative ANOVA test of the seven developmental stages. The analysis yielded a subset of 3333 proteins (Fig. 5A); a *k*-means clustering assay divided them into 9 exclusive clusters based on their abundance outlines (Fig. 5B) (full list of 3333 proteins according to their *k*-means cluster appear in Supplementary Table S1). Among these, clusters 1 and 9 consist of 1077 proteins whose levels significantly declined along fruit development (*p* value = 0.026 and 0.0179, respectively), while the 793 proteins in clusters 5 and 7 displayed a substantial increase in abundance along fruit development (*p* value = 0.0051 and 0.0061, respectively), particularly from 30 DAF onward, when periderm tissue starts to form below the cracked skin surface (Fig. 5C). We, therefore, focused on the latter subset of proteins, as we expected it to include proteins that function in metabolic pathways, regulatory mechanisms and other processes essential for the formation of lignosuberized periderm tissue.

We subjected proteins belonging to the functional categories of these 793 proteins to Gene Ontology (GO) analysis and plotted them under a network-based structure (Fig. 5D). As predicted, we found proteins belonging to fatty acid biosynthesis, as well as aromatic amino acid, phenylpropanoid, suberin, lignin and indolalkylamine pathways; their corresponding encoding genes had been previously tightly linked with periderm tissue formation (Fig. 5D). The identification of an additional network composed of proteins participating in cellular detoxification responses against oxidative stress indicated that the structural changes in skin that occur during the formation of lignosuberized periderm tissue may induce this type of stress (Fig. 5D).

### Proteomics data highlight the accumulation of proteins associated with lipid metabolism, cell wall modification, vesicle-mediated trafficking and stress responses, during the formation of periderm

Among the 793 proteins included in clusters 5 and 7, we initially focused on proteins involved in the metabolism of components tightly linked with the initiation of a lignosuberized periderm tissue. We were able to detect 53 putative proteins involved in the metabolism of phenylpropanoids, suberin, cutin, lignin, lignans, lipids and flavonoids. Several putative key phenylpropanoid proteins were at low abundance in 10 and 20 DAF skin, but over-accumulated at 30 DAF onward, when lignosuberized periderm tissue was detected below the cracked skin. These included PHENYLALANINE AMMONIALYASE1 (PAL1), which catalyzes the first step in the pathway in which phenylalanine undergoes deamination to yield *trans*-cinnamic acid; 4-COUMARATE:COENZYME A LIGASE 2 and 3 (4CL2 and 4CL3), which catalyze the ATP-dependent formation of the CoA thioester 4-coumaroyl CoA; HYDROXYCINNAMOYL-COA SHIKIMATE/QUINATE HYDROXYCINNAMOYL TRANSFERASE (HCT), which catalyzes the transfer of the *p*-coumaroyl group to shikimate as well as the transfer of a caffeoyl moiety back to Co-enzyme A; CAFFEOYL COENZYME A *O*-METHYLTRANSFERASE1 (CCOAOMT1), which synthesizes feruloyl-CoA from caffeoyl-CoA, the first methyl transfer reaction in the pathway; CINNAMOYL COA REDUCTASE 1 (CCR1), which catalyzes the synthesis of hydroxycinnamaldehydes from the hydroxycinnamoyl-CoA thioesters; CAFFEATE *O*-METHYLTRANSFERASE1 (COMT1), which methylates 5-hydroxy-coniferaldehyde and 5-hydroxyconiferyl alcohol to yield sinapaldehyde and sinapyl alcohol, respectively; and an NADPH-CYTOCHROME P450 REDUCTASE2 (ATR2), which catalyzes the first oxidative step in the pathway ( [15, 18]; Table 1).

**Table 1:** List of proteins identified in clusters 5 and 7 that are associated with the metabolism of phenylpropanoids, suberin, cutin, lignin, lignans, lipids and flavonols. Blue colors represent low abundances while red colors represent high abundances.

$\includegraphics{\bwartpath uhac092t1}$

^1^Cucumber protein ID was extracted using the Cucurbit Genomics Database (CuGenDB; http://cucurbitgenomics.org/);

^2^Arabidopsis homolog ID, gene description, homology percentages and E-values was extracted using the tools embedded in The Arabidopsis Information Resource (TAIR; https://www.arabidopsis.org/)

Concomitantly, a subset of putative proteins with known functions in the suberin biosynthetic pathway considerably accumulated following the appearance of periderm tissue in 30 DAF samples, but were hardly detected in 10 and 20 DAF skin. This group contained KETOACYL COA SYNTHASE2, which encodes for a fatty acid elongase condensing enzyme involved in the biosynthesis of aliphatic suberin (KCS2; [19]); GLYCEROL 3-PHOSPHATE ACETYLTRANSFERASE5 (GPAT5), which is essential for the synthesis of glycerol-based suberin [20]; ALIPHATIC SUBERIN FERULOYL-TRANSFERASE (ASFT), which generates aromatic suberin monomers by using ferulate derivatives derived from the phenylpropanoid pathway [21, 22]; FATTY ACID REDUCTASE1 (FAR1), which generates suberin long-chain fatty alcohols [23, 24]; cytochrome P450s CYP86A1 and CYP86B1, which are involved in the formation of $\omega $-hydroxy long-chain fatty acid suberin monomers [25, 26, 27]; ATP-BINDING CASSETTE G transporters ABCG2 and ABCG6, which were demonstrated to localize in the plasma membrane and transport suberin monomers to their deposition sites [28]; and LIPID TRANSFER PROTEIN1 (LTP1), which was shown to participate in cell wall suberization of Arabidopsis crown galls [29]. Interestingly, ACYL CARRIER PROTEIN4 (ACP4) and GPAT8, two proteins involved in cuticle development and assembly [22, 30], exhibited relatively constant accumulation patterns along fruit development (Table 1).

Suberin, lignin and lignan metabolism tightly depends on the supply of precursors from the core phenylpropanoid pathway and was previously associated with periderm tissue formation in reticulated melon fruit [3]. Here, we detected 23 putative proteins that function in these two pathways. Three LACCASES (LAC3, LAC4 and LAC12) and five PEROXIDASES (PRX17, PRX52, PRX53, PRX64 and PRX72), which oxidize monolignols in the lignin pathway [31], were absent in 10 DAF and started to accumulate from 20 DAF onward to various degrees. Comparable accumulation patterns were detected for CAFFEOYL SHIKIMATE ESTERASE (CSE), which participates in monolignol biosynthesis [32, 33]; BLUE COPPER BINDING PROTEIN (BCB), a glycosylphosphoatidylinositol-anchored protein that regulates lignin biosynthesis [34]; CYTOCHROME B5 ISOFORM B (CYTB5B), an obligate electron shuttle protein for syringyl lignin biosynthesis [35]; CASPARIAN STRIP MEMBRANE DOMAIN-LIKE PROTEINS CASPL1D1 and CASPL4D1, which are involved in the lignin polymerization machinery [36]; and UCLACYANIN1 and 2 (UCC1 and UCC2), which were recently associated with lignification of the root endodermis [37]. Lignan biosynthesis requires the coupling of two coniferyl alcohols by laccases or peroxidases, with the aid of DIRIGENT PROTEINS, to form pinoresinol, followed by the activity of PINORESINOL REDUCTASEs (PRRs), an NADPH-dependent reductase that is considered pivotal for lignans’ structural diversity [38]. Indeed, the levels of six DIRIGENT-like proteins as well as PRR1, PRR2, and PHENYLCOUMARAN BENZYLIC ETHER REDUCTASE1 (PCBER1), which catalyzes NADPH-dependent reduction of 8–5′ linked lignans, were very low at 10 DAF, but increased from 20 DAF onwards, particularly in the later developmental stages, i.e. 50, 60 and 70 DAF (Table 1).

As SPAD consists of lipidic components linked with glycerol, we expected to find changes in the abundance of proteins involved in lipid metabolism. Indeed, a subset of putative proteins displayed comparable accumulation patterns to those involved in lignosuberization processes, suggesting that these changes are linked with the appearance of periderm tissue. These included LIPASE1 (LIP1), which is involved in triacylglycerol degradation [39]; BCCP-LIKE PROTEIN2 (BLP2), which functions in fatty acid biosynthesis [40]; PATATIN-LIKE PROTEIN2 (PLP2), which catalyzes fatty acid release [41]; PASTICCINO (PAS2), a VLC hydroxyl fatty acyl CoA dehydratase [42]; LIPOXYGENASE1 and 3 (LOX1 and LOX3), two ubiquitous enzymes that catalyze the dioxygenation of polyunsaturated fatty acids [43]; and GLYCOSYLPHOSPHATIDYLINOSITOL-ANCHORED LIPID PROTEIN TRANSFER 7 (LTPG7), which is predicted to be involved in lipid transport. Markedly, the abundance of all these proteins was significantly higher in 70 DAF skin compared to the earlier stages, suggesting a possible shift in lipid metabolism at this particular stage, when the skin cracks and the periderm tissue is prominent (Table 1).

In agreement with the accumulation of several flavonoids identified in our metabolite profiling assays along fruit development, we detected an increase in abundance in three putative proteins with roles in flavonoid biosynthesis, further strengthening a link between this biochemical group and periderm tissue formation. These included UDP-GLUCOSYLTRANSFERASEs UGT73B2 and UGT 85A5, shown to function in the flavonoid pathway [44, 45]; and β-GLUCOSIDASE15 (BGLU15), which acts on flavonol 3-*O*-β-glucoside-7-*O*-α-rhamnosides [46] (Table 1).

It is apparent that lignosuberized periderm formation is accompanied by substantial catalytic activities of acyl transferases, α/β-, glycosyl- and SGNH-hydrolases, GDSL esterases/lipases and lipoxygenses, as many of these proteins were found in clusters 5 and 7. The levels of these proteins gradually rose from 30 DAF onward (Supplementary Table S1). Other proteins in these cluster participate in cell-wall remodeling and organization, such as pectate lyases, pectin methylesterases, pectin acetylesterases, expansins, arabinosyltransferases, ceremidases; all exhibited similar accumulation patterns to those described above, with a particular increase in abundance in 70 DAF samples (Supplementary Table S1).

Another group of proteins of high interest are those involved in vesicle-mediated trafficking. These included four COPII-associated vesicle transport proteins from the rough endoplasmic reticulum (ER) to the Golgi apparatus, EMP24GOLGI, CYTOPLASMATIC BODIES (CYB), P24BETA2 and TRANSDUCIN; three SNARE proteins that facilitate the transport of vesicles from the Golgi apparatus to the plasma membrane and/or vacuole; VESICLE-ASSOCIATGED MEMBRANE PROTEIN724 (VAMP724), VESICLE TRANSPORT V-SNARE11 (VTI11) and TOMOSYN11 (TYN11); and docking and tethering factor RAB GTPASE HOMOLOG A1F (RABA1F) (Supplementary Table S1).

Finally, we detected a subset of proteins involved in response to oxidative stress, such as SUPEROXIDE DISMUTASE (SOD), GLUTATHIONE *S*-TRANSFERASE14 (GST14) and six PEROXIDASEs; they accumulated with the appearance of lignosuberized periderm tissue, at 30 DAF. Twelve HEATSHOCK PROTEINs were found to highly accumulate only in 60 and 70 DAF samples (Supplementary Table S1).

## Discussion

### Periderm tissue formation in the Sikkim cucumber is coordinated by spatiotemporal interplays between cutin, suberin and lignin pathways

Our assays demonstrated that the periderm tissue of the Sikkim cucumber skin is made primarily from suberized and lignified cells, as previously shown in specialized periderm tissues of potato tubers [16], tomato [47], kiwifruit [48], apple [49], melon [3], and sand pear [50]. The first signs of a brownish coating and small cracks above the skin surface were visible already at 20 DAF fruit; histological observations further validated the initiation of periderm tissue at this developmental stage. Using histochemical staining, we showed that lignin accumulates in periderm cells below the cuticle at 20 DAF, while fluorescent staining and electron microscopy demonstrated that suberin initiates its deposition at 30 DAF. In agreement with this evidence, our proteomic survey inferred that lignin-related proteins accumulate already at 20 DAF while proteins involved in different aspects of suberin metabolism remain virtually undetected at 20 DAF but highly accumulate at 30 DAF and onward. The two polymers also differed in their spatial deposition configurations within the periderm tissue, as suberin was confined exclusively to periderm cells while lignin was detected not only in periderm layer but also at the outermost cuticle conical cells. Markedly, these deposition patterns diverge from the ones we previously detected in the periderm tissue of reticulated melon fruit, where suberized cells localized solely to the upper cell layer of the periderm, while the lower periderm cells underneath appeared to be lignified [3]. Altogether, these findings propose that the formation of periderm tissue in the Sikkim cucumber is coordinated by a spatiotemporal interplay between suberin and lignin, and apparently depends on the regulation of both pathways.

Additional metabolic interplay during periderm formation involves the cutin and suberin pathways. The levels of the three major cutin monomers, C_16_ ω-hydroxyacid, C_16_–10,16-dihydroxyacid and C_18_–9,10,18-trihydroxyacid, along with coumaric and caffeic acids, were relatively similar in green skin and skin coated only with a brownish layer with no visible cracks. This implies that at this particular stage, the brown layer, even though it features the initial accumulation of typical suberin monomers, has no effect on the levels of cutin. The accumulation of suberin was mostly associated with the cracked skin regions at the later fruit developmental stages of 50, 60 and 70 DAF, rather than the stage when a brownish layer coats the skin, at 30 and 40 DAF. These include typical suberin monomers from the biochemical groups of VLCFAs, fatty alcohols, $\omega $-hydroxyacids and α,$\omega $-diacids. The latter stages, however, also featured a substantial decrease in the abundance of all the five cutin monomers mentioned above, suggesting tight regulation between the cutin and suberin pathways during periderm tissue development. Our results are in accordance with previous reports in apple [1, 49], tomato [51] and pear [52], indicating that fruit skin suberization often comes at the expense of cutin biosynthesis.

### Metabolic repertoires of lignosuberized periderm tissue during fruit skin development

Thus far, the majority of research on fruit skin suberization has focused on identifying specific changes in suberin and cutin profiles using targeted metabolite profiling approaches. Our multi-omics approach augmented by statistics enabled us to treat the data as a whole in order to disentangle the metabolic repertoire of lignosuberized periderm tissue during fruit skin development. Apart from changes in the level of suberin and cutin monomers described above, our data points to several aromatic components that were highly associated with periderm tissue formation and thus represent part of the SPPD repertoire. These include ferulic acid, di-ferulyol triglucoside, lavandoside, robustaside B, phenylpropionic acid, hydroxybenzoic acid hexose, vanillic acid, and benzyl alcohol hexose pentose. Some of these compounds were also found in the metabolic profile of reticulated melon fruit periderm tissue, which featured lower levels of flavonoid derivatives, inferring metabolic shifts in between branches of the phenylpropanoid pathway [3]. In the Sikkim cucumber, however, we detected higher levels of flavonoids such as naringenin hexose, naringenin chalcone hexose, luteolin 7-*O*-β-glucoside and kaempferol hexose deoxyhexose, surmising that the aromatic moiety of skin periderm tissue differs even between two related cucurbitaceous species.

While the cuticle protects the epidermal cell layer of the fruit, other membrane lipids protect the internal cells and organelles. In plants, the plasma membrane is made primarily of PCs and PEs; MDGDs, DGDGs and PGs represent the plastidic membrane fraction and their function is tightly associated with photosynthesis; PIs are relatively minor in cellular membranes, yet play important roles during lipid signaling; and DAGs and TAGs serve as major reserve storage components [53]. The fruit skin lipidomic profiles we obtained clearly show a constitutive reduction in the levels of virtually all PCs, PEs, PGs, MDGDs, DGDGs and PGs, once the lignosuberized periderm tissue, a brownish coating and cracks appear above the fruit surface, at 20 DAF. As from this developmental stage onwards the skin is rapidly coated with the brownish corky layer that later on undergoes massive cracking, photosynthesis is expectedly reduced, as are its associated lipid moieties. DAGs and TAGs displayed comparable reduced amounts towards fruit maturation, yet it is hard to directly link these accumulation patterns to the formation of lignosuberized periderm, as these components tend to degrade in skin tissues upon fruit ripening [54]. Sterols were largely associated with cuticular waxes and have been identified in surface extracts of various plant species [55, 56, 57, 58]. Hence, we could attribute the reduction in sterols to the lower synthesis of cuticle components and the overall rewiring of fatty acids toward the synthesis of suberin building blocks at the expense of cutin monomers. Unlike almost all lipid components identified in our profiling, ceramides displayed a significant rise in abundance, which fitted well with the formation of lignosuberized periderm tissue at 20 DAF, suggesting a possible link between the two processes. Ceramides are integral membrane components that can also act as signaling molecules to regulate plant development and stress responses [59]. We, therefore, postulate that ceramides might play important roles in reinforcing the membranes of cells building the lignosuberized periderm tissue. We do not rule out that ceramides may also function as important signaling molecules in the development of the fruit skin and/or in stress responses that occur during these processes, as reflected by the significant accumulation of stress-related proteins. Nevertheless, there is no evidence that links the biosynthesis of ceramides to the formation of periderm tissue, and the roles of these lipidic components during fruit skin development requires further attention.

### A proteomic catalogue of a lignosuberized periderm tissue

Transcriptional profiling of periderm tissue was performed on russeted fruit skin of apple [60] and sand pear [50], on potato tuber skin [61], on the bark of poplar [62] and cork oak [63] and on reticulated skin of melon fruit varieties [3]. Albeit these studies provide imperative insight into the core transcriptional networks involved periderm tissue formation, the expression of a gene does not necessarily reflect the accumulation of protein encoded by this said gene. But a few recent studies used proteomics to isolate proteins involved in the process of periderm tissue formation. For instance, an integrated transcriptomics and proteomics approach was used to investigate the molecular mechanisms controlling apple russeting [64]. Proteomics was used to understand the ABA stimulation of wound suberization in kiwifruit skin [65], while two recent studies exploited multi-omics approaches to reveal regulatory and stress-responsive networks operating in the russet fruit skin of sand pear [50, 66].

Our proteomics investigation showed an accumulation trend of proteins involved in phenylpropanoid, suberin and lignin pathways in connection with the appearance of skin cracks and periderm tissue formation in the Sikkim cucumber fruit. We identified putative proteins involved in the major reactions of the core phenylpropanoid pathway, from early steps of deamination to ring and side-chain modifications and methyl-transfer reactions. In the same way, the formation of lignosuberized periderm tissue was accompanied by massive accumulation of putative proteins involved in different aspects of suberin and lignin pathways. These included proteins executing the synthesis of suberin aliphatic and aromatic monoacylglycerols (ASFT and GPAT5, respectively), $\omega $-hydroxylation of monomers (CYP86A1 and CYP86B1), formation of alcohols (FAR1), and transport of suberin building blocks (ABCG2, ABCG6 and LTP1; [67]); along with typical lignin proteins such as LACCASEs, PEROXYDASEs, CASPs, and UCLACYANINs. Correspondingly, peroxidase activity and gene expression were detected in reticulated skin of melon fruit [3, 68]. In addition, CASP family homologs were identified in suberized periderm of potato tubers and melon fruit, as well as in a suberin gene expression signature dataset generated by multispecies co-expression analyses [3, 51, 61]. While the contribution of these three pathways to periderm tissue formation are in line with many other reports from different species, the secondary metabolites, lignans, gained much less attention with respect to these processes. The Sikkim cucumber periderm tissue featured massive accumulation of several DIRIGENT-like proteins, PRRs and PCBER1. In agreement with this evidence, lignan compounds and transcripts were identified in the lignosuberized periderm of reticulated melon fruit [3] and in suberized green cotton fibers [69]. The lignan pinoresinol was detected in cork extracts of outer bark tissues of Douglas fir tree (*Pseudotsuga menziesii*) [70]. The composition of lipids in Flax (*Linum usitatissimum* L.) seeds also contains suberin monomers and lignans [71]. These accumulative literature data, along the presence of lignans in the reticulated skin of the Sikkim cucumber detected herein, suggest that lignans might act as essential structural backbones of the suberin polymer and/or lignosuberized periderm tissue. Further effort, however, is required to determine a possible link between the suberin lamellae and lignans.

As in reticulated melon skin, the structural rearrangements in the epidermal cell layer and cuticle of the cracked skin of the Sikkim cucumber were accompanied by an expected rise in abundance of dozens of proteins involved in cell-wall remodeling and organization. Our results also point to the over-accumulation of stress-related proteins, particularly those involved in oxidative stress (SOD1, GST14 and PEROXIDASEs). Indeed, previous studies reported on stress-responsive genes in cork oak [72, 73], as well as in native and wound-healing potato tuber skin [74, 75, 76]. Put together, these evidences suggest that the establishment of a lignosuberized periderm tissue is a coordinated structural and metabolic process associated with the induction of stress.

Finally, the dataset composed of proteins belonging to clusters 5 and 7 may be considered to be a protein catalogue of lignosuberized periderm tissue. For instance, enzymes possessing different catalytic activities were highly associated with the appearance of periderm and the accumulation of suberin and lignin, including acyl transferases, α/β hydrolases, glycosyl hydrolases, SGNH hydrolases/esterases, GDSL esterases/lipases and lipoxygenases. Proteins belonging to these families were previously proposed to play roles in suberization and lignification processes. Therefore, the functional portrayal of these candidates might shed light on their possible part in the assembly and polymerization of suberin and lignin polymers. In the same way, the subset of vesicle trafficking-related proteins identified here may provide a suitable platform with which to study the elusive transport mechanism by which suberin and/or lignin monomers are distributed from their biosynthesis sites to their deposition sites.

## Materials and methods

### Plant material and growth conditions

Plants of the Sikkim cucumber (*C. sativus* var. *sikkimensis*) were grown under controlled greenhouse conditions with periodic fertilization supplement of 20:20:20 N:P:K. Fruit were harvested during development at 10 day intervals: 10, 20, 30, 40, 50, 60, and 70 days after fertilization (DAF). Fruit skin tissues were either freshly dissected for cutin and suberin analyses using GC–MS, immersed in fixation buffers for histology and microscopy, or stored for further metabolomics, lipidomics and proteomics analyses.

### Microscopy

All procedures and protocols associated with histological sectioning, histochemical staining of cell walls (toluidine blue) and lignin (phloroglucinol-HCL) and fluorescent staining of suberin (fluorol yellow 088), light and fluorescent microscopy, and electron microscopy (SEM and TEM), are similar to those previously described by [3].

### Metabolomics, lipidomics and proteomics

For metabolite profiling of cutin and suberin monomers, skin discs at diameter of 1 cm were freshly harvested from fruit at the seven investigated developmental stages, and de-lipidated for consecutive 2 weeks in methanol:chloroform buffer (v/v; GC–MS purity grade), and fully dehydrated for 3 days in a desiccator containing activated silica-gel beads. Transesterification of skin samples was achieved via the addition of 4 mL of boron trifluoride:methanol (Sigma-Aldrich; 99.8%) and incubation at 70°C for 16 hours. Transesterification was stopped using the addition of saturated NaHCO3/water into the samples. All further steps of phase separation, the addition of *n*-dotriacontane (C_32_) alkane internal standard, sample derivatization with pyridine (Sigma-Aldrich; 99.8%) and *N*,*O*-bis(trimethylsilyl)trifluoroacetamide (Sigma-Aldrich; GC–MS purity grade) reagents, and GC–MS apparatus and running parameters, are similar to those previously described by [3].

For metabolomics of semi-polar compounds, 100 mg of frozen homogenized powder originated from skin discs of fruit at the seven investigated developmental stages, were extracted with 80% methanol UPLC purity grade and 0.1% formic acid (1:3 w/v), vortexed vigorously followed by a sonication step for 15 min. Clear supernatants were achieved via 15 min of centrifugation at 20800 *g* and finally passed through 0.22-μm PTFE filters. Samples were then run in an UPLC-HRMS apparatus with similar parameters as previously described by [77] (list of 34 identified semi-polar metabolites appear in Supplementary Table S2).

For lipidomics, 100 mg of frozen homogenized powder originated from skin discs of fruit at the seven investigated developmental stages, was extracted using 1 mL of pre-cooled (−20°C) homogenous methanol:methyl-*tert*-butyl-ether (MTBE) (1:3 v/v), spiked with the internal standards: 0.1 μg/mL 17:0 PC (Avanti Polar Lipids, Inc. #850360P), 0.4 μg/mL 17:0 PE (Avanti Polar Lipids, Inc. #830756P), 0.15 nmol/mL Ceramide/Sphingoid Internal Standard Mixture I (Avanti Polar Lipids, Inc. RF0000000785 / LM4-030A) and 0.0267 μg/mL d5-TG Internal Standard Mixture I (Avanti Polar Lipids, Inc. RF0000000780 / LM4-025A). Samples were then ultra-sonicated for 30 min at RT. Phase separation was achieved by the addition of 500 μL of UPLC purity grade water:methanol (3:1 v/v) and centrifugation at 13000 rpm and 4°C for 5 min. The upper organic phase was transferred to a new tube. The lower aqueous phase was re-extracted with additional MTBE, and the combined organic phases were dried in a Speed Vac Concentrator and stored at −80°C until use. The dried lipid extracts were re-solved in 300 μL mobile phase B prior to analysis via a UPLC Waters Acquity instrument connected in-line to a Synapt HDMS detector (tandem quadrupole/time-of-flight mass spectrometer) equipped with an Electrospray Ionization (ESI) source. Compounds were separated on a UPLC BEH C8 1.7 μm, 2.1 mm X 100 mm column with mobile phase A: 45% DDW, 55% Acetonitrile (ACN): 2-Isopropanol (IPA) (7:3) + 1% 1 M Ammonium acetate and 0.1% Acetic acid and mobile phase B: ACN: IPA (7:3) + 1% 1 M Ammonium acetate and 0.1% Acetic acid with a flow rate of 0.4 mL/min and column temperature of 40°C with the following gradient: 0 min solvent A 100%, solvent B 0%; 1 min solvent A 100%, solvent B 0%; 12 min solvent A 25%, solvent B 75%; 15 min solvent A 0%, solvent B 100%; 21 min solvent A 0%, solvent B 100%; 21.5 min solvent A 100%, solvent B 0%; 25 min solvent A 100%, solvent B 0%. Mass spectrometric detection in positive ionization mode was performed at the range of 50–1200 *m/z* in centroid mode using MS^E^, recording spectra with collision energy 4 eV in channel one, and spectra with a collision energy ramp from 15 to 35 eV in channel two. All spectra were lock mass calibrated using leucine enkephalin. For identification of lipid species, raw data files were converted into mzXML format files, organized into respective sample groups, and subjected to peak-picking process using the XCMS software [78]. The XCMS output was processed with the MSBox R-package for matching detected mass spectra to in-house lipid database. All returned potential identifications were validated manually using MassLynx software (Waters) (list of 165 identified semi-polar metabolites appear in Supplementary Table S3).

Lastly, proteomics analyses of skin discs of fruit at the seven investigated developmental stages was achieved using similar protocols as recently described by [77]. For the reference cucumber proteome, we used the most updated version of the cucumber genome assembly available in the Cucurbit Genomics Database (CuGenDB; http://cucurbitgenomics.org/).

## Statistical analyses

Bar graphs were compiled using the GraphPad Prism v8.0.1, a versatile statistics tool purpose-built for scientists (https://www.graphpad.com/), and significance was calculated according to the two-way ANOVA test of *p*-value <0.05 (Fig. 3); one-way ANOVA test, based on an adjusted *p*-value (FDR) cutoff of <0.01 and post-hoc Tukey’s HSD (Fig. 4); or a comparative ANOVA (FDR <0.05) (Fig. 5). The number of biological replicates is mentioned in the corresponding figure legend of each experiment. PLS-DA of 201 significant-changed metabolites along the isolation of VIPs was performed using MetaboAnalyst v5.0, a comprehensive tool suite for metabolomic data analysis (http://metaboanalyst.ca/; [79]), following data log_10_ transformation and pareto scaling (mean centered and divided by the square root of SD of each variable) manipulations. Following the isolation of 3333 significantly-changed proteins along fruit development, a *k*-means clustering assay divided them into 9 exclusive clusters based on their abundance outlines. Curve graphs representing the accumulation trend of proteins included in each of these clusters were generated using the GraphPad Prism v8.0.1 including calculated slopes, *R^2^* and *p* values for every cluster. Finally, Gene Ontology (GO) analyses of the 793 proteins belonging to clusters 5 and 7 were performed using the tools embedded in the Cucurbit Genomics Database (CuGenDB; http://cucurbitgenomics.org/), and presented as a network constructed using Cytoscape v3.9.0, an open-source platform for visualizing complex networks (https://cytoscape.org/).

## Acknowledgments

The authors would like to thank Ms. Natalie Page for proofreading the manuscript. We also thank the Adelis Foundation, Leona M. and Harry B. Helmsley Charitable Trust, Jeanne and Joseph Nissim Foundation for Life Sciences, Tom and Sondra Rykoff Family Foundation Research and the Raymond Burton Plant Genome Research Fund for supporting the activity in Asaph Aharoni laboratory. Asaph Aharoni is the incumbent of the Peter J. Cohn Professorial Chair.

## Author contributions

G.C.A commenced and took part in all experiments in the frame of this project; Y.D. and N.S. analyzed the metabolomics data; U.H. analyzed the lipidomics data, Y.K. and E.A. analyzed the proteomics data; G.N., O.M. and E.M. assisted in the cultivation of the Sikkim cucumber and technical procedures; A.A and H.C. planed and designed the project; G.C.A, A.A. and H.C. wrote the manuscript.

## Data Availability

The authors declare that all the data supporting the findings of this study are available within the paper and its supplemental information files.

## Conflict Interests statements

The authors declare that they have no competing financial interests or personal relationships that could have influenced the work reported in this paper.

## Supplementary data

Supplementary data is available at *Horticulture Research * online.

## Supplementary Material

Web_Material_uhac092
