# Supplementary material for: The metabolic and proteomic repertoires of periderm tissue in skin of the reticulated Sikkim cucumber fruit
Source: Hortic Res. 2022 Apr 22;9:uhac092. doi: 10.1093/hr/uhac092 (PMC9160728; doi:10.1093/hr/uhac092)
Supplement: Web_Material_uhac092 [file web_material_uhac092.zip › Supplemental Figure S1.pdf]

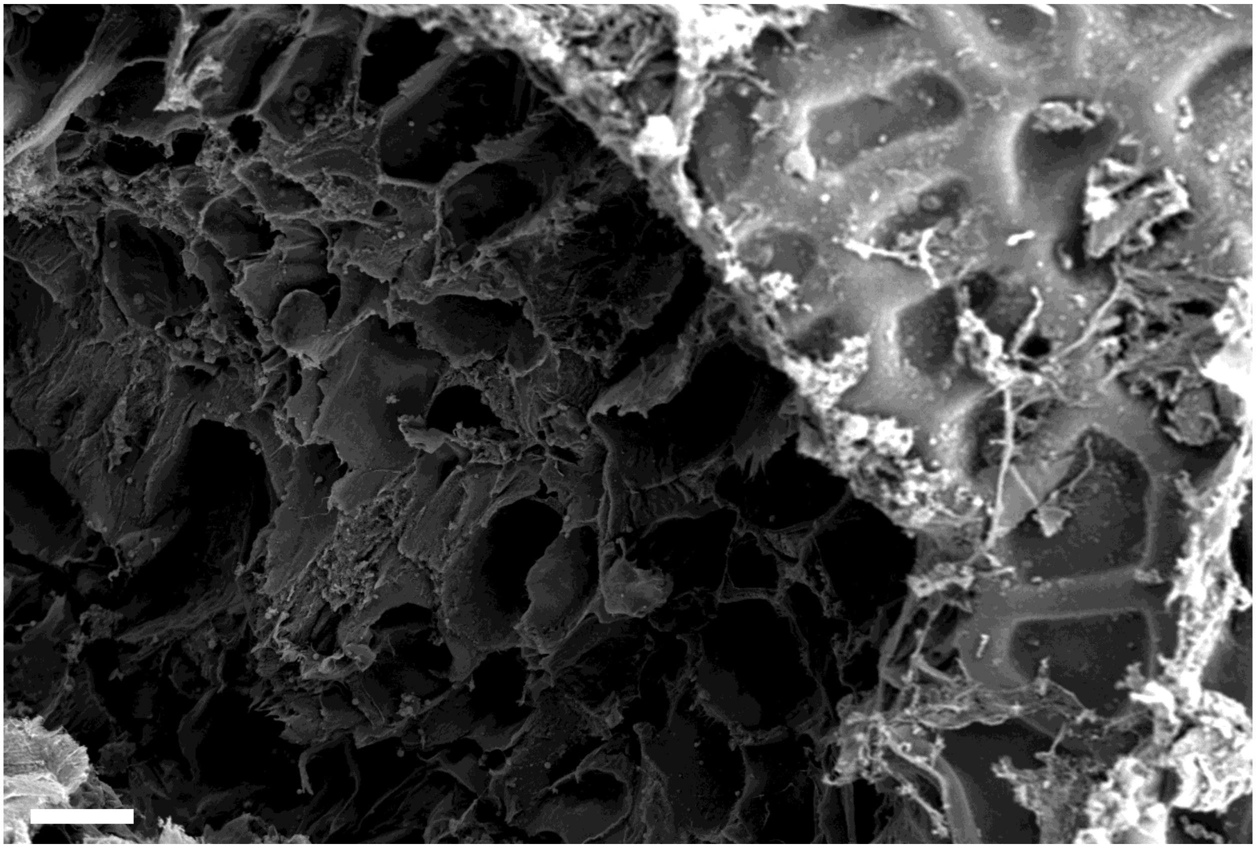

**Supplemental Figure S1. Cross-section SEM micrographs reveal typical organized phellem cell layers in periderm tissues of the Sikkim cucumber.** Scale bar = 10 µm.
